# Anti-inflammatory cytokine and angiogenic factors levels in vitreous samples of diabetic retinopathy patients

**DOI:** 10.1371/journal.pone.0194603

**Published:** 2018-03-27

**Authors:** Teresa Tsai, Sandra Kuehn, Nikolaos Tsiampalis, Minh-Khoa Vu, Vinodh Kakkassery, Gesa Stute, H. Burkhard Dick, Stephanie C. Joachim

**Affiliations:** Experimental Eye Research Institute, Eye Hospital, Ruhr-University Bochum, Bochum, Germany; Massachusetts Eye & Ear Infirmary, Harvard Medical School, UNITED STATES

## Abstract

Evaluation of cytokines in patients with diabetic retinopathy (DR) is important for the identification of future additive or alternative treatment options. Therefore, vitreous samples were obtained from patients with DR and patients with macular hole or macular pucker (control group) during 23-gauge-vitrectomy (n = 17/group). The levels of three pro-inflammatory (IL-1ß, IL-6, IFN-γ) and pleiotropic cytokines (IL-2, IL-4, IL-13) as well as VEGF, VEGF-A, and PGF were measured using an enzyme linked immunosorbent assay (ELISA). IL-1ß (p = 0.02) and IFN-γ (p = 0.04), two of the three tested pro-inflammatory cytokines, were elevated in the DR patients, while IL-6 (p = 0.51) level was comparable in both groups. Moreover, in DR samples, a trend towards an IL-13 down-regulation (p = 0.36) was observable. The IL-2 (p = 0.62) and IL-4 (p = 0.78) levels were comparable in both groups. All analyzed angiogenetic factors were up-regulated in DR patients (VEGF: p<0.001; VEGF-A: p = 0.002; PGF: p<0.001). The up-regulation of angiogenetic factors underline their importance in DR development. However, the interaction of the other cytokines showed an interesting pattern. Pro-inflammatory cytokines were also up-regulated, which could be evidence for inflammation processes in the diabetic retina. Furthermore, it seems that a counter response of immunomodulatory cytokines is in an initial process, but not strong enough to regulate the processes. Therefore, to support these anti-inflammatory mechanisms might be additive treatment option in the future.

## Introduction

Diabetes mellitus (DM) is a systemic disease and one of the most important causes of mortality and health-system costs in the world [1]. Risk factors that lead to DM are population growth and aging as well as an increasing prevalence of obesity and physical inactivity. Therefore, the number of people with DM worldwide is expected to approximately double between 2000 and 2030 [2].

Diabetic retinopathy (DR) is the most frequent microangiopathy complication of diabetes and currently the main cause of visual impairment and blindness among working-age adults in western countries [3–5]. The prevalence of DR is related to the duration of diabetes. Usually, in the first years after diagnosis of type 1 DM, the prevalence of DR is very low, but after more than 20 years most diabetics develop a retinopathy [6]. People with type 2 DM can be directly affected by DR at diabetes diagnosis, due to years of undiagnosed diabetes. After 20 years of type 2 DM, around 60% of these patients have some form of DR [6, 7]. Therefore, the personal and socioeconomic costs of this condition are very high.

DR is a progressive condition with microaneurysms and small hemorrhages, which lead to retinal ischemia, permeability, and neovascularization [8, 9]. Damage of neurons through ischemic processes plays an important role in DR. Ischemia, due to capillary blockage, leads to non-perfusion of this region of the retina. Few hours after ischemia, inflammation as well as an activation of retinal glial cells occurs [10, 11]. In turn, these activated glia cells release several different cytotoxic substances, which are responsible for blood-barrier break-down and induce leucocyte, glia as well as vascular dysfunction [12, 13]. All these cellular responses are signs for inflammatory processes that seem to play a role in ischemia-induced retinal injuries, like DR. [14].

Beside inflammatory processes, neovascularization is a hallmark of DR pathogenesis. The vascular endothelial growth factor (VEGF) contributes to angiogenesis, influences vascular permeability and is postulated as the key regulator of DR [15].

In the last years, several factors besides VEGF moved into the focus of DR progression. For example, pro-inflammatory cytokines and chemokines, like interleukin IL-1ß, IL-6, and interferon-γ (INF-γ), which are released by activated glia cells, were up-regulated in retinae of diabetic animals [16–19]. Therefore, they seem to be important for DR development. Interestingly, also in DR patients, cytokine and chemokine alterations seem to play a crucial role. This is reflected in the fact that it was possible to detect an increase of endothelin-1 (ET-1), TNF-a, and IL-6 in vitreous samples of proliferative type 2 DM patients via ELISA assay [20]. In another study, a decrease of tyrosine phosphorylation of vitreous proteins was detectable via immunoblot assay in type 2 DM patients after vitrectomy [21]. These identified factors increase the evidence that inflammation processes act as critical contributors to the DR development [19, 22]. Nevertheless, at the moment, not all important cytokine/chemokine members of these processes and the signaling mechanism involved in this response are yet clear [19].

Moreover, the placental growth factor (PGF) acts synergistically with VEGF. Therefore, PGF plays a role in the development of ocular angiogenic disorders and was found to be increased in the vitreous samples of patients with non-proliferative and proliferative DR, but was nondetectable in control samples [23]. Thus, the idea arises, to use this protein and to identify new proteins as key biomarkers for DR [7, 24].

A number of potential DR therapies have been tested in clinical trails. Currently, an anti-VEGF monotherapy has the most effective treatment success rates and leads to the best visual acuity. However, many treatments are necessary during the first few months and possibly years to achieve a successful disease treatment. Addionally, about 30% of DR patients fail to respond to the initial anti-VEGF treatment [15, 22]. Therefore, the question arises which other factors are involved in the pathogenesis of DR and can be used for disease treatment.

Therefore, the aim of this study was to identify possible vitreous cytokine alterations due to DR. These findings could help to develop future additive treatment options. Especially, knowledge about the interaction and regulation of angiogenetic factors, pleiotropic and inflammatory cytokines is important for a successful therapy.

## Material and methods

### Subjects and vitreous sampling

Ruhr-University ethic committee approval (Bochum, Germany; approval number: 15–5363) was obtained for the vitreous sample collection, the tenets of the Declaration of Helsinki were observed. Written informed consent was obtained from all patients. Patients were categorized after clinical entry examination based retinopathy stages from the Early Treatment in Diabetic Retinopathy Studies (ETDRS). For every patient clinical data, including mean age, sex, eye, diabetes type, DR stadium as well as HbA1c, were collected (summarized in Table 1, individual data are stated in S1 Table).

**Table 1:** Clinical patient data for both groups: Controls and diabetic retinopathy (DR) patients. Y = Year; M = male, F = female; OD = right eye; OS = left eye. DR stages are: 1 = mild to moderate NPDR, 2 = severe NPDR, and 3 = PDR.

	controls	DR patients
**Mean Age±SD (y)**	74.6±8.7	63.1±12.2
**Sex (M/F)**	9/8	5/12
**Eye (OD/OS)**	7/10	9/8
**Diabetes type (1/2)**	---	6/11
**DR stage±SD**	0	1.59±0.5
**HbA1c±SD**	---	7.1±0.7

Undiluted vitreous samples were obtained from 17 patients with DR undergoing vitrectomy and patients undergoing vitrectomy for macular hole or macular pucker (control group). The vitreous samples were collected prospectively and undiluted by transconjunctival 23-gauge-pars-plana-vitrectomy (1ml/patients), immediately frozen and stored at -80°C until analysis.

### Inclusion criteria

Prior to sample collection, patients were divided in three groups. There, the classification was based on the modified Arlie-House classification, also used in the Early Treatment in Diabetic Retinopathy Studies (ETDRS): Controls (patients with macular hole or macular pucker undergoing vitrectomy), mild non-proliferative diabetic retinopathy (mild NPDR) and proliferative diabetic retinopathy (PDR).

### Exclusion criteria

Patient with an age under 21, a presence of other ocular diseases other than age-related cataract, a current or past anti-VEGF therapy (systemic or local treatment), previous vitrectomy, other ocular surgery three month before, laser therapy three month before, steroid medication three month before (including systemic, topical, and intravitreal application), and glaucoma have not been included in this study.

### Measurement of cytokines in vitreous samples

All samples of the study were measured at the same time by the same investigator with the standard preparation to avoid inter-user or plate variability (n = 17/group). Levels of pro-inflammatory (IL-1ß, IL-6, and INF-γ) and anti-inflammatory cytokines (IL-2, IL-4, and IL-13) in vitreous samples were quantified using commerially available enzyme immunassay kits (ELISA; R&D systems, Minneapolis, MN; ebioscience, San Diego, CA). Additionally, the level of vascular endothelial growth factor (total VEGF and VEGF-A; R&D systems, Minneapolis, MN; ebioscience, San Diego, CA) as well as the PGF level (R&D systems, Minneapolis, MN) was measured by ELISA. Each assay was performed according to the manufacturer’s instructions. For each measurement samples were diluted (for the exact dilution factor of each ELISA see Table 2) using sample diluent buffer just prior to the assay. All measurements were performed on a microplate reader (AESKU Reader with Gen5 ELISA Software, AESKU. DIAGNOSTICS, Wendelsheim, Germany).

**Table 2:** Applied ELISA assays including company, catalogue number, used dilution and references.

Protein	Company	Catalogue number	Dilution	Reference
IL-1ß	R&D Systems	DLB50	1:2	[49]
IL-2	R&D Systems	D2050	1:2	[50]
IL-4	Bender/ebioscience	BMS225/2	1:2	[51]
IL-6	R&D Systems	D6050	1:50	[52, 53]
IL-13	Bender/ebioscience	BMS231/3	1:2	-
INF-γ	Bender/ebioscience	BMS228	1:5	[51]
VEGF A	Bender/ebioscience	BMS277/2	1:2; 1:5	-
VEGF	R&D Systems	DVE00	1:2; 1:5	[54]
PGF	R&D Systems	DPG00	undiluted	[55]

### Statistics

All statistical analyses were performed using the commercial predictive analytic Statistica program (Version 13; Dell, Tulsa, OK). Significance in cytokine concentration between study and control groups was calculated using the non-parametric Mann-Whitney U test. The subgroups (no DR, mild NPDR, PDR) groups were compared using Kruskal Wallis test with Dunn’s multiple comparison test. A *p*-value less than 0.05 was considered statistically significant. Data were recorded as the dot plots with mean.

## Results

In total, 34 vitreous samples were analyzed. The patients in the control group had a mean age of 74.6±8.7 years and the DR patients a mean age of 63.1±12.2 years. Therefore, the control group was significantly older than the DR patients. This is due to the younger age of disease onset in patients of type I DM. In the DR group, 6 patients had DM type I and 11 patients had DM type II. In sum, all DR patients exhibited an average DR stage of 1.59±0.5 (Table 2). Moreover, laboratory values, which provide diabetic indications, were collected. Regarding gender and side ratio of the operated eye no significant differences were detectable between both groups (Table 2). An overview of all patient data and their accompanying cytokine concentration is deposited in S1 Table.

### Increased pro-inflammatory cytokine concentrations

**Fig 1:**
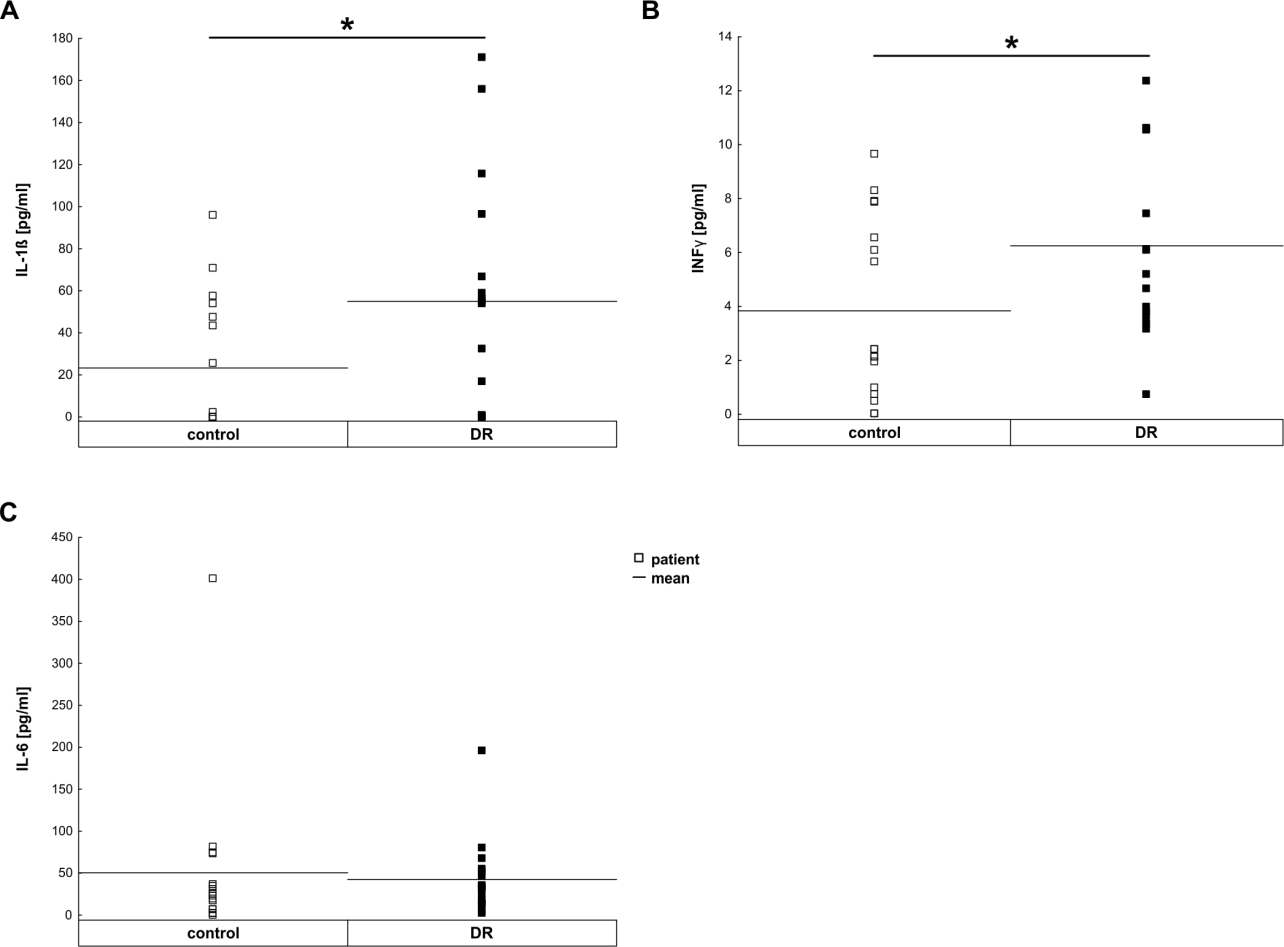
Increased pro-inflammatory cytokine levels in vitreous samples. **A.** The pro-inflammatory cytokine IL-1ß (p = 0.02) was significantly up-regulated in compassion to the controls. **B.** INF-γ (p = 0.04) was also significantly up-regulated. **C.** In contrast, IL-6 expression level was the same in DR and control patient (p = 0.51). Each symbol depicts an individual patient. The horizontal bar indicates the mean cytokine concentration per group. *: p<0.05.

The proinflammatory cytokines, IL-1ß, IL-6, and INF-γ, were detectable in all vitreous samples from patients with DR and the non-diabetic control patients (Fig 1 and Table 3). The expression levels of IL-1ß (control: 23.28±7.65 pg/ml, DR: 54.98±13.03 pg/ml; p = 0.02, Fig 1A) and INF-γ (control: 3.83±0.8 pg/ml, DR: 6.25±0.84 pg/ml; p = 0.04; Fig 1B) were significantly up-regulated in DR samples. Whereas, the IL-6 expression level of DR patients (42.29±10.94 pg/ml) was comparable to the level of the control group (50.40±22.76 pg/ml; p = 0.51; Fig 1C). Dividing the DR patients in two subgroups–mild NPDR and PDR- abolished the difference in the expression level of IL-1ß and INF-γ based on the relatively small number of patients in this pilot study (S1A–S1C Fig).

**Table 3:** Cytokine concentration (pg/ml) in vitreous samples of control and diabetic retinopathy (DR) patients measured via ELISA. Significant p-values are in bold.

	Cytokine concentrations in vitreous samples		
	control	DR patient	p-value
**IL-1ß**	23.28 ± 7.65	54.98 ± 13.03	**0.02**
**IL-2**	5.12 ± 1.45	6.32 ± 1.52	0.62
**IL-4**	0	0.14 ± 0.14	0.78
**IL-6**	50.40 ±22.76	42.29 ± 10.94	0.51
**IL-13**	3.12 ± 0.19	2.85 ± 0.14	0.36
**INF-γ**	3.83 ± 0.80	6.25 ± 0.84	**0.04**
**VEGF**	12.11 ± 3.03	731.20 ± 222.72	**0.002**
**VEGF-A**	6.16 ± 4.00	1525.73 ± 461.31	**<0.001**
**PGF**	0.19 ± 0.54	73.00 ± 142.68	**<0.001**

### Minimal regulation of pleiotropic immunomodulatory cytokines

**Fig 2:**
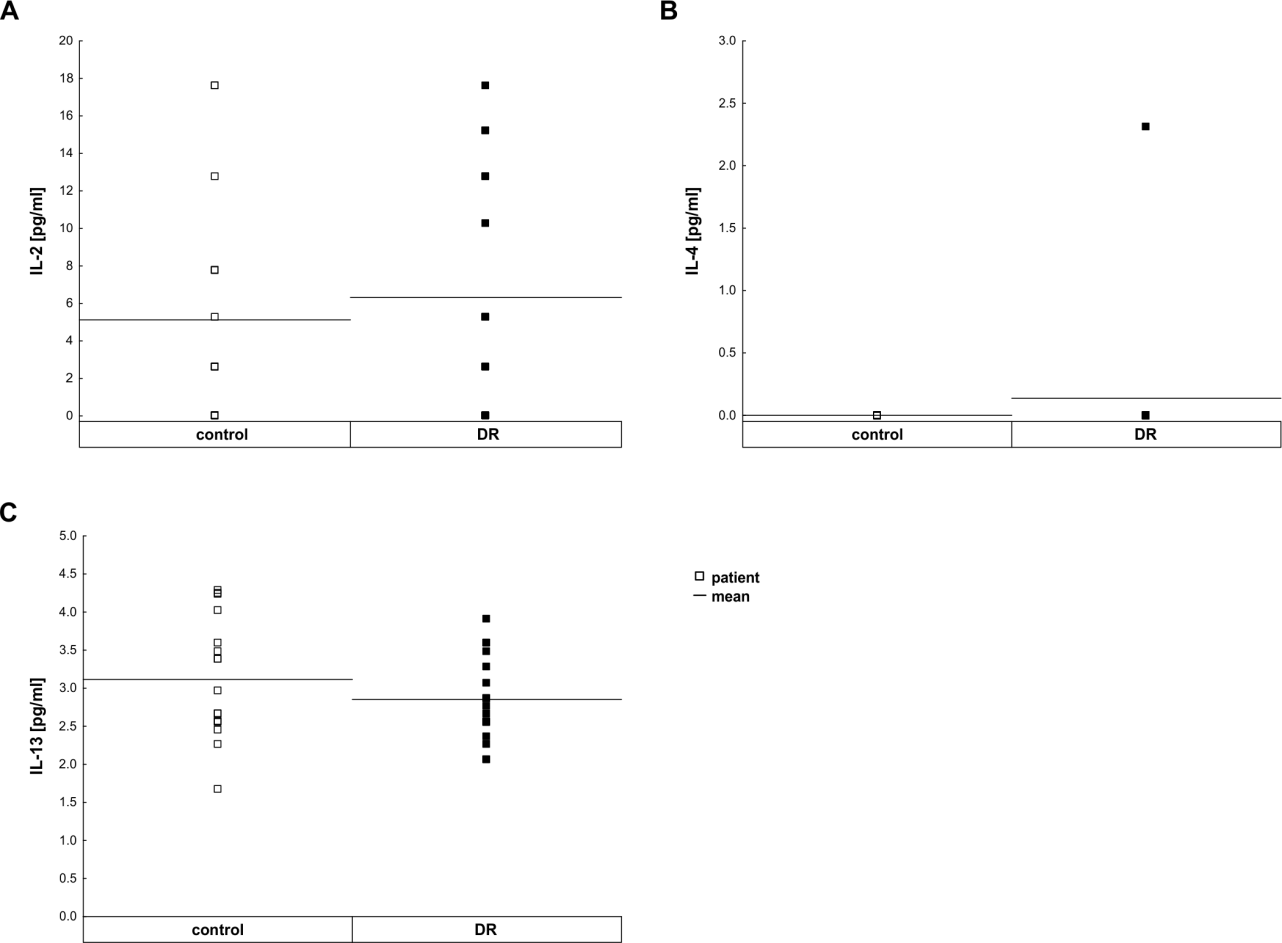
Contribution of pleiotropic immunomodulatory cytokine levels in vitreous samples. **A.** A difference in IL-2 (p = 0.62) level was not measurable between control and DR patients. **B.** There was no difference in IL-4 level between control and DR patients (p = 0.78). **C.** A trend for the down-regulation of IL-13 level (p = 0.36) was noted in DR patients. Each symbol depicts an individual patient. The horizontal bar indicates the mean cytokine concentration per group.

Regarding the pleiotropic cytokines, a slight trend towards an IL-13 (control: 3.12±0.19 pg/ml, DR: 2.85±0.14 pg/ml; p = 0.36; Fig 2C) down-regulation was observed in DR samples. In contrast, the IL-2 level (control: 5.12±1.45 pg/ml, DR: 6.32±1.52 pg/ml; p = 0.62; Fig 2A, Table 3) and IL-4 level (control: 0 pg/ml, DR: 0.14±0.14 pg/ml; p = 0.51; Fig 2B) were comparable in DR and control group. Significant differences in the expression level of IL-4, IL-13 or IL-2 were also not detectable after dividing the DR patients in two subgroups, mild NPDR and PDR (S1D–S1F Fig).

### Strong stimulation of angiogenic factors

**Fig 3:**
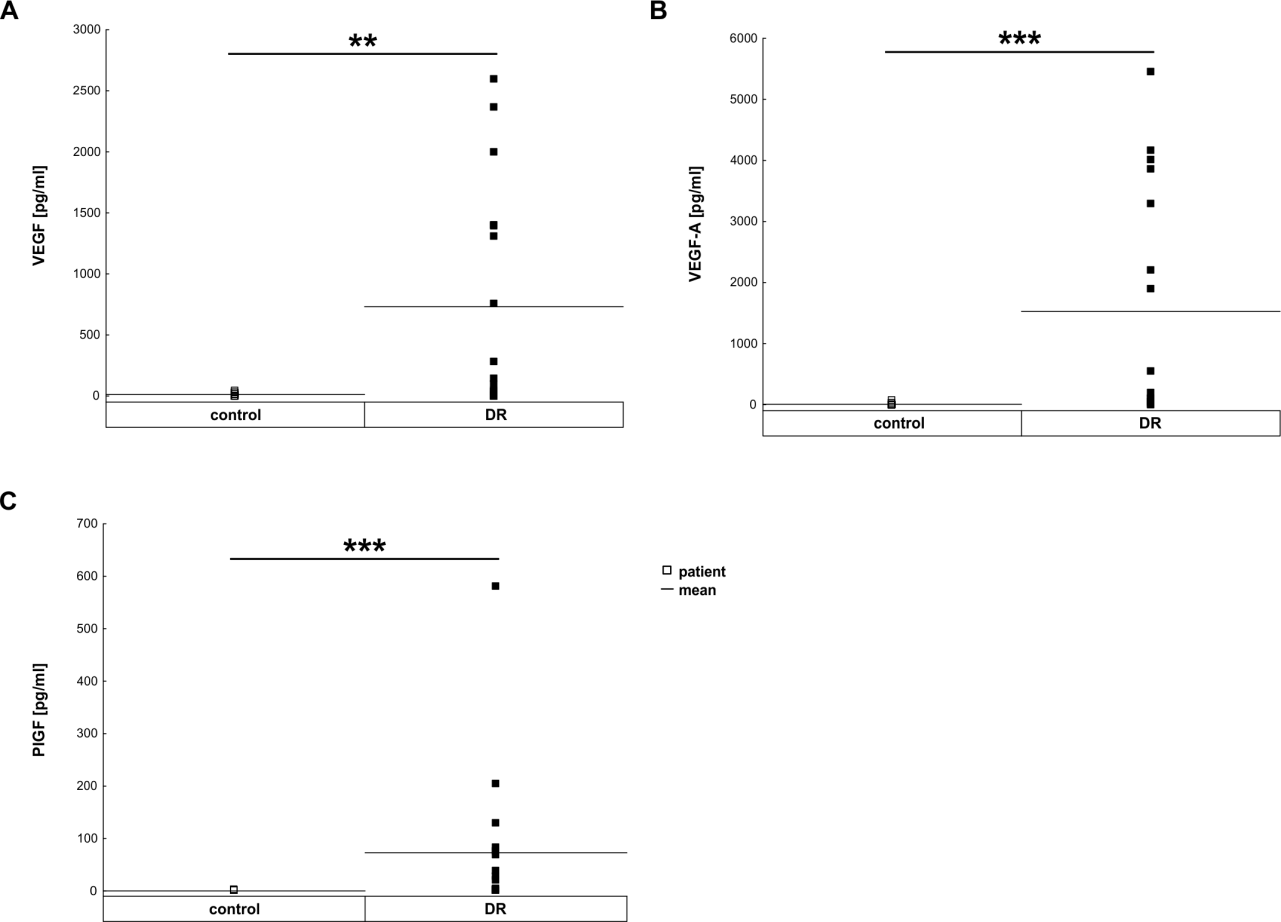
Up-regulation of different angiogenic factors in vitreous samples. **A.** Total VEGF was significantly up-regulated in vitreous samples of DR patients (p = 0.002). B. VEGF-A was also significantly up-regulated in DR patients (p<0.001). **C.** Additionally, PGF was upregulated in DR samples compared to controls (p<0.001). Each symbol depicts an individual patient. The horizontal bar indicates the mean cytokine concentration per group. **: p<0.01, ***: p<0.001.

Compared to control patients, vitreous samples of DR patients revealed an up-regulation of all analyzed angiogenetic factors (Fig 3 and Table 3). The mean level of total VEGF in vitreous samples of DR patients (731.20±222.72 pg/ml) was significantly higher than in control patients (12.11±3.03 pg/ml; p = 0.002; Fig 3A). Also, the level of VEGF-A was significantly up-regulated in DR patients (1525.73±461.31 pg/ml) in comparison to the controls (6.16±4.00 pg/ml p<0.001; Fig 3B). Additionally, the level of PGF was up-regulated in DR patients (control: 0.19±0.54 pg/ml; DR: 73.00±142.68 pg/ml p<0.001; Fig 3C). This significant upregulation in the angiogenic factors was even observable after subdividing the DR patient into two groups (S1G–S1I Fig).

## Discussion

In the current study, we evaluated alterations in vitreous samples due to DR. In the course of this, we noted a significant increase in pro-inflammatory cytokine concentrations and a minimal regulation of pleiotropic immunomodulatory cytokines in the vitreous of DR patients. Additionally, DR patients exhibited a significant up-regulation of angiogenic factors.

DR is a sight-threatening condition, which seems to evolve to an increasingly common phenomenon in the next decades due to the worldwide increase in DM. DR is a progressive condition with microaneurysms and small hemorrhages which lead to retinal ischemia, permeability, and neovascularization [25]. VEGF contributes to angiogenesis and influences vascular permeability. Anti-VEGF therapies can lead to a substantial improvement in visual function and prevent disease progression targeting the vascular dysfunction. Although, anti-VEGF applications improved the DR therapy, a frequent administration is needed and still a lot of patients experience a significant visual loss under therapy [3, 15]. Interestingly, also other candidates besides VEGF may contribute to the disease process. Therefore, the aim of this prospective study was to identify new possible cytokine alterations due to DR.

For a long time, DR has not been recognized as an inflammatory disease, based on the fact that the retina is a tissue with immune privilege. Nevertheless, typical clinical signs of DR, such as edema and neovascularization, are hallmarks of inflammation. Although the retina is an immune privileged place, up-regulation of iCAM caused by VEGF signal pathway in DR patients [26] is a sign for the contribution of leucocytes [27]. Therefore, the potential roles of inflammatory mediators in DR became the subject of investigation [14, 21, 22, 28]. Consequently, we analyzed three pro-inflammatory cytokines (IL-1ß, IL-6, and INF-γ) in vitreous samples of patients. The three pro-inflammatory cytokines were detectable in the vitreous samples of all analyzed subjects. Moreover, IL-1ß and INF-γ expression levels were significantly elevated in DR patients, whereas the level of IL-6 was unaffected in the DR group. In line with our results also other groups identified elevated levels of IL-1ß and its activator molecule caspase-1 in vitreous of patients with proliferative diabetic retinopathy [29, 30]. Moreover, an up-regulation of IL-1ß could be identified in retinas of rats and mice, where diabetes was induced by streptozotocin injections [29, 31, 32]. The proinflammatory cytokine IL-1ß is known to induce vascular dysfunction as well as cell death via an increase of endothelia permeability and caspase-3 activation [29, 32]. Both processes occur during DR progression. Interestingly, IL-1ß inhibition by canakinumab, which is a human monoclonal antibody targeted at interleukin-1ß, is already by used for the treatment of other inflammatory diseases, like familial cold autoinflammatory syndrome and Muckle–Wells syndrome [33–35]. It has no cross-reactivity with other members of the interleukin-1 family, including interleukin-1 alpha and therefore it could be a good candidate for DR treatment in the future.

Besides IL-1ß, we also found an up-regulation of the pro-inflammatory cytokine INF-γ in the vitreous samples of DR patient. INF-γ is classically part of the helper T-cell class 1 (Th1) cytokine response, which activates pro-inflammatory M1 macrophages or microglia and B-cells. These are criteria for inflammatory innate and adaptive immunity. There are some hints in the literature about an up-regulation of INF-γ in the retina of diabetic rats, but an elevated INF-γ expression level in patients has not been reported so far [36]. Interestingly, another study on nephropathy in type 1 DM identified a correlation of INF-γ level with haemostatic biomarkers [37]. For different Th-1-mediated autoimmune diseases, including rheumatoid arthritis, multiple sclerosis, and corneal transplant rejection, an immunomodulatory therapy represents a treatment option [38]. Rheumatoid patients exhibited a significant improvement, if they were treated with INF-γ [39]. Nevertheless, the role of INF-γ in several autoimmune diseases, like multiple sclerosis, has remained as an enigmatic paradox for several years, since IFN-γ seems to play a protective and destructive role [40]. Therefore, an INF-γ therapy for DR-treatment holds great promise, but the exact role of INF-γ in DR, more precise in the different DR stages, has to be investigated in detail before.

Moreover, other studies could detect an up-regulation of IL-6 in the vitreous of DR patients [41], which is in contrast to our results. Nevertheless, these studies identified a correlation between the increase of the IL-6 expression level and the severity as well as progress of DR. The minor number of PDR patients (Table 1) in our study could be an explanation for our missing IL-6 up-regulation. Another explanation for our missing IL-6 up-regulation, could be the cross-talk between IL-1β and IL-6 signaling, more precisely the inhibitory action of IL-1β on IL-6 signaling [42]. Consequently, for an unequivocal evidence for the IL-6 function in DR, a larger number of DR patients, a more uniform distribution of DR stages and the interaction of IL-1β and IL-6 in vitreous samples should be analyzed in the future.

Besides the pro-inflammatory cytokines, also the level of three pleiotropic cytokines (IL-4, IL-2, and IL-13) was analyzed. In regard to IL-13 a trend towards a lower release was observed in DR samples. The levels of IL-2 and IL-4 remained unchanged. These three cytokines represent Th-2 based cytokines. Regarding the IL-4 level, our results differ from a previous survey, which identified an elevated IL-4 level in the vitreous sample of PDR patients, which could be interpreted as a compensatory response aiming to reduce inflammatory processes in DR or as a sign of the humoral immune response. The fact that we did not analyzed only PDR patients but also mild NPDR patient samples could be an explanation for the missing significant difference in our study. Furthermore, besides IL-4, also IL-13 can act anti-inflammatory by downregulating pro-inflammatory cytokines, like TNF-α, IFN-γ, and IL-12 [43]. Therefore, the observable trend of IL-13 downregulation could be a hint for missing anti-inflammatory activities in DR patients and should be analyzed in more detail since it might offer a new treatment option.

The concentration of angiogenic factors was also measured in the vitreous samples of DR and control samples. In the vitreous samples of DR patients an up-regulation of VEGF, VEGF-A, and PFG was measurable in comparison to control patients. The up-regulation of these factors is in agreement with several previous reports [23, 44, 45]. Since 2004, it is known that VEGF is up-regulated in the vitreous of patients with proliferative DR [46]. The ELISA, which only detects VEGF-A in our study, also represented up-regulated VEGF-A levels in our DR patients. Therefore, our ELISA results of VEGF and VEGF-A underline the correctness of our data and the importance of the angiogenetic factors- especially VEGF-A, in DR treatment.

In addition to VEGF and VEGF-A, we also analyzed the amount of PGF. Like VEGF, PGF plays an important role in angiogenesis [47] Interestingly, PGF not only activates its own signaling via VEGFR, but also enhances VEGF signaling by displacing VEGF-A from VEGFR-1 to VEGFR-2. Subsequently PGF amplifies VEGFR-2 signaling [48]. In line with our results, increased levels of PGF have also been found in the vitreous of proliferative-DR patients [23], which may indicate that PGF is involved in the progression of DR.

Therefore, our results of the angiogenic factors underline, that targeting multiple VEGF family members may provide therapeutic strategies for the DR treatment in the future.

In conclusion, our study indicates that pro-inflammatory cytokines and angiogenetic factors are associated with inflammation and angiogenesis, which contribute to the pathogenesis of DR. Currently, anti-angiogenesis therapy via VEGF-treatment is the most successful DR treatment. In other diseases, for example rheumatoid arthritis or multiple sclerosis, immunomodulatory therapies represent a new and effective treatment options. Thus, both anti-inflammatory and anti-neovascularization agents could be used, possibly simultaneously, in the DR treatment in the future. Moreover, additional identifications of cytokine alterations in DR as well as a correlation between DR stages and cytokine alterations might lead to additive treatment options.

## Supporting information

S1 Fig Cytokine distribution in different patient subgroups. All analyzed patients are grouped into three categories (no DR, mild NPDR, PDR). **A-C.** The level of the pro-inflammatory cytokines IL-1ß, INF-γ, and IL-6 did not differ significantly between the different patient subgroups based on the relatively small number of patients in each subgroup. **D-F.** Also, the pleiotropic cytokines IL2, IL-4, and IL13 were comparable in all groups. **G.** In regard to the angiogenic factors, VEGF was significantly upregulated in PDR samples compared to no DR (p<0.001) and mild NPDR (p<0.01). **H.** Comparable effects were seen for VEGF-A, a significant upregulation was noted in the PDR group compared to the no DR (p<0.001), but not to the mild NPDR group (p>0.05). **I.** Also, a significant upregulation of PGF was observed in in the PDR group compared to the no DR (p<0.001) and the mild NPDR group (p<0.05). Each symbol depicts an individual patient. The horizontal bar indicates the mean cytokine concentration per group. *: p<0.05, **: p<0.01, ***: p<0.001.
(TIF)

S1 Table Demographic information (age, gender, patient group, type of DM, DR subgroup, HbA1c level) and cytokine levels (pg/ml) in vitreous samples of control (white) and DR patients (grey; DME patient is labeled in red). (DOCX)
