# Supplementary figures and images for: Anti-inflammatory cytokine and angiogenic factors levels in vitreous samples of diabetic retinopathy patients
Source: PLoS One. 2018 Mar 27;13(3):e0194603. doi: 10.1371/journal.pone.0194603 (PMC5870958; doi:10.1371/journal.pone.0194603)

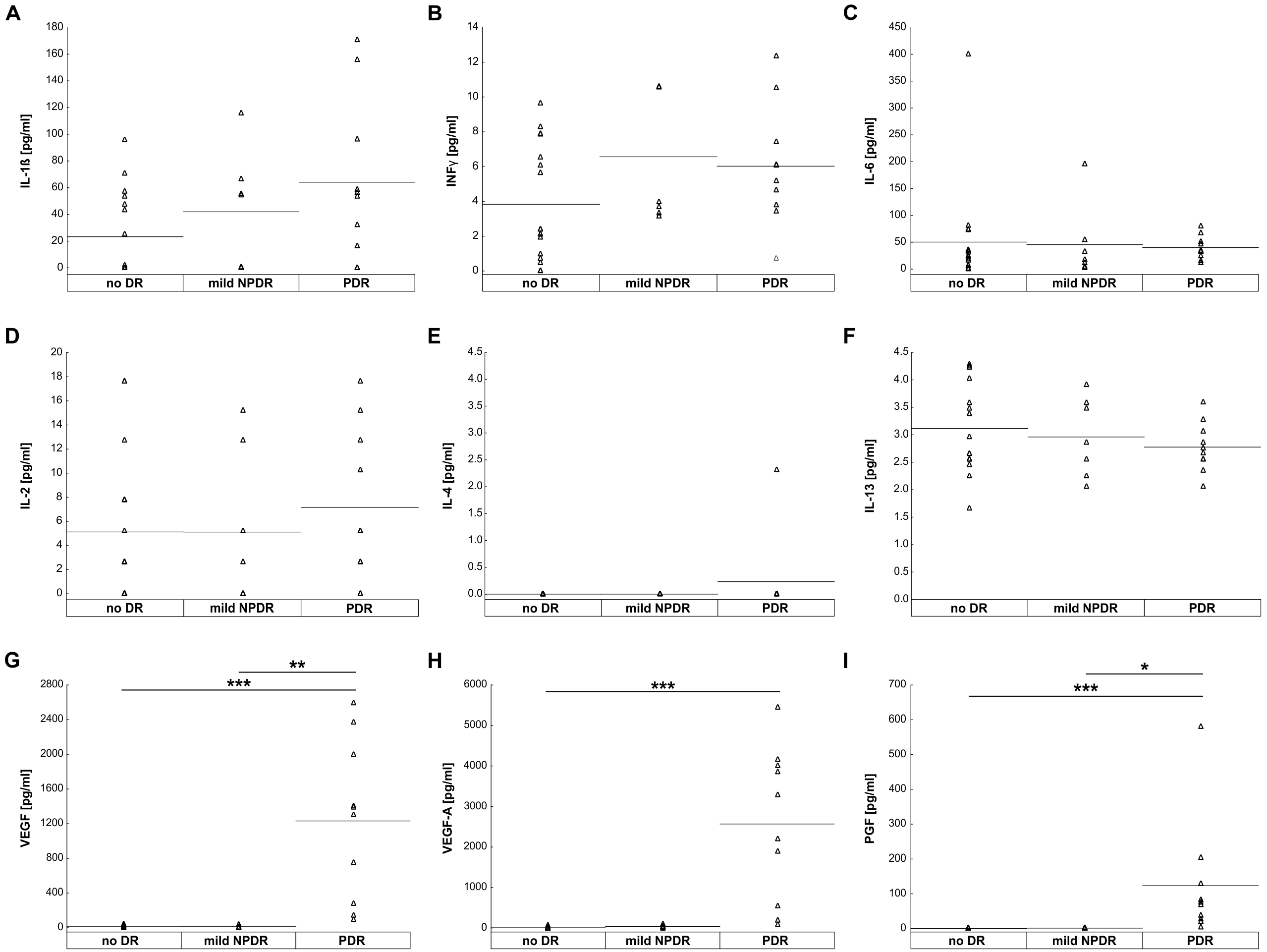

Supplement: S1 Fig — All analyzed patients are grouped into three categories (no DR, mild NPDR, PDR). A-C. The level of the pro-inflammatory cytokines IL-1ß, INF-γ, and IL-6 did not differ significantly between the different patient subgroups based on the relatively small number of patients in each subgroup. D-F. Also, the pleiotropic cytokines IL2, IL-4, and IL13 were comparable in all groups. G. In regard to the angiogenic factors, VEGF was significantly upregulated in PDR samples compared to no DR (p<0.001) and mild NPDR (p<0.01). H. Comparable effects were seen for VEGF-A, a significant upregulation was noted in the PDR group compared to the no DR (p<0.001), but not to the mild NPDR group (p>0.05). I. Also, a significant upregulation of PGF was observed in in the PDR group compared to the no DR (p<0.001) and the mild NPDR group (p<0.05). Each symbol depicts an individual patient. The horizontal bar indicates the mean cytokine concentration per group. *: p<0.05, **: p<0.01, ***: p<0.001. (TIF) [file pone.0194603.s001.tif]
