# Supplementary material for: Anti-inflammatory cytokine and angiogenic factors levels in vitreous samples of diabetic retinopathy patients
Source: PLoS One. 2018 Mar 27;13(3):e0194603. doi: 10.1371/journal.pone.0194603 (PMC5870958; doi:10.1371/journal.pone.0194603)
Supplement: S1 Table — (DOCX) [file pone.0194603.s002.docx]

S1 Table: Demographic information (age, gender, patient group, type of DM, DR subgroup, HbA1c level) and cytokine levels (pg/ml) in vitreous samples of control (white) and DR patients (grey; DME patient is labeled in red).
